# Development and Validation of the COVID-19 Worries and Fears Scale

**DOI:** 10.3389/ijph.2022.1604600

**Published:** 2023-01-09

**Authors:** Esther Cuadrado, Alicia Arenas, Manuel Moyano, Martina La Gamma

**Affiliations:** ^1^ Department of Psychology, University of Cordoba, Córdoba, Spain; ^2^ Maimonides Biomedical Research Institute of Cordoba (IMIBIC), Cordoba, Spain; ^3^ Department of Social Psychology, Universidad de Sevilla, Seville, Spain; ^4^ Department of Psychology, University of Florence, Florence, Italy

**Keywords:** COVID-19, worries, fears, scale development, anxiety, protective behavior

## Abstract

**Objectives:** How individuals perceive the risk of COVID-19 influences their mental health and protective behaviors. Therefore, the development of an instrument to capture COVID-19-related worries and fears is relevant. This study aims to develop and validate the CoV-WoFe to measure COVID-19-related worries and fears.

**Methods:** An online questionnaire was completed by 593 participants during Christmas 2020 and by 328 participants during Summer 2021, from which 88 participants formed a longitudinal sample.

**Results:** Analyses confirmed a robust adjustment for consistency over time and a gender-invariant bifactorial structure. Factor 1 represented worry about the health consequences of COVID-19 and Factor 2 represented the perceived physiological symptoms associated with fear of COVID-19. Construct validity was evidenced by: the expected relations between the CoV-WoFe and other theoretically related constructs; the serial mediating role of both dimensions in the relationship that security values establish with protective behaviors against COVID-19 and with anxiety; and the expected gender differences in the Cov-WoFe.

**Conclusion:** The CoV-WoFe represents a short, valid, reliable, gender-invariant tool that is easy to apply in both the health professional and research context to assessCOVID-19-related worries and fears, which are variables of relevance for spread of the virus and for mental health.

## Introduction

The COVID-19 pandemic has generated a context of uncertainty all over the world [1]. The success of policies to slow down the rapid transmission of the virus relies in part on accurate perceptions of the risk factors [2]. By knowing how individuals perceive the risk of COVID-19, healthcare providers and policymakers could design appropriate programs to slow down the pandemic. Therefore, the main aim of this study—to develop and validate a brief instrument to capture the COVID-19-related worries and fears (the COVID-19 Worries and Fears Scale—the COV-WoFe)—seems relevant.

## Theory and Components of COVID Risk Perception

The relationship between risk perception and safe behavior has been strongly established in the literature on occupational health and safety and public health [3]. Different theoretical models of cognitive orientation—such as the Theory of Reasoned Action [4–6]—have tried to describe the process through which people face a risky situation and what leads them to adopt preventive measures. Ferrer et al. [3] developed the TRIRISK model of risk perception in which deliberative, affective and experiential components are distinguished, although other studies have combined the experiential and affective component in one unique component [7–9]. In this sense, many studies conceptualize risk perception as a bidimensional construct, with both a cognitive and an affective component [9, 10]. When considering the impact on protective behaviors, several studies have shown that the affective component—feeling the risk—has greater relevance than the cognitive component—analyzing the likelihood of the risk [11]. In fact, in the pandemic context, different studies have corroborated the greater relevance of the worries and fears related to COVID-19 in comparison to the perceived probability of becoming infected for predicting different protective behaviors [9, 12, 13]. Thus, the development and validation of a scale that taps into the affective component of COVID-19 risk perception seems to be of relevance in the pandemic context.

During a pandemic, many people exhibit fear- and anxiety-related distress responses; however, the emotional aspect of risk perception has not been sufficiently explored [14]. Thus, we developed the CoV-WoFe, covering the emotional dimension of risk [15, 16] and focusing on two different aspects: (a) worry about the health consequences of COVID-19 (CoV-Wo), representing worry about catching the virus and its health implications; and (b) the fear of COVID-19 (CoV-Fe), representing how individuals present uncontrollable anxiety and fear related to COVID-19. We posit the hypothesis (H1) that the structure of the CoV-WoFe will show two dimensions (CoV-Wo and CoV-Fe) consistently over time.

### Gender Differences in COVID-19-Related Worries and Fears

Women and men are differentially affected by COVID-19 and the pandemic [17], thus there is a need to carry out COVID-19 studies from a gender perspective [17, 18]. Accordingly, we will explore the invariance of the CoV-WoFe to ensure the validity of the scale for men and women. Moreover, many studies have suggested that gender can play a significant role in risk perception, with women tending to rate risk higher than men [19, 20]. Therefore, we would expect women to show higher levels of worries and fears over COVID-19 than men (H2).

### COVID-19-Related Worries and Fears Over Time

To better manage the pandemic, understanding people’s worries and fears about the virus over time is important [21]. However, few studies have analyzed the affective part of risk perception and the factors involved longitudinally [9, 22].

With the extremely high infection rate and relatively high mortality at the end of 2020, individuals began worrying about COVID-19. Fear also may have amplified the perception of damage itself, which could have influenced reactions and protective behaviors over time. Therefore, taking a several-month lapse, it is expected that in periods in which the infection rates are decreasing, and the information given by the authorities and media is more positive, the worries and fears of individuals should be lower than in other periods in which the infection rates are increasing and the information given is alarming (H3).

### Factors Associated With COVID-19-Related Worries and Fears

Evaluation of the external validity of the CoV-WoFe supposes the assessment of relationships with other constructs potentially related to worries and fears of COVID-19. To hear about COVID-19 being a cause of death is an indirect experience of a COVID-19 hazard that supposes higher worry and fear levels [8]. Thus, we expect that being or knowing people who are especially vulnerable to COVID-19 and knowing loved ones who have died from COVID-19 will be factors related to the worry and fear over the disease.

Personal values oriented toward self-protection may have an impact on how people perceive risks. In this sense, people oriented toward self-protection, who give importance to their security, adopted more protective behaviors in the pandemic context [23, 24] to protect themselves from COVID-19 contagion. Accordingly, people particularly worried about self-protection will perceive higher COVID-19 risks.

Another important factor related to risk perception in times of COVID-19 is skepticism. Similar to climate change and vaccination, skepticism towards COVID-19 may have consequences for how people perceive risk and the intention to protect themselves [25]. Thus, a negative relationship between skepticism regarding COVID-19 and the CoV-WoFe is expected.

Risk perception induces responses to cope with crises, exerting significant influence on decisions and behaviors [26] and puts people in an anxious state, leaving them to take precautions such as protective behaviors to avoid more serious consequences [27]. Moreover, as explained earlier, several studies have found a relationship between the emotional component of risk perception and protective behaviors against COVID-19 [8, 9, 12, 13]. Thus, we expect to find a positive relationship between the CoV-WoFe and the intention to adopt protective behaviors to avoid COVID-19 contagion, and the protective behaviors themselves, and also between the CoV-WoFe and anxiety [27].

Therefore, we expect (H4) that the CoV-WoFe will show relationships with: (a) direct experience with COVID-19 (being or living with a COVID-19-vulnerable person and having loved ones who have died from COVID-19 will be positively related with worries and fears of the disease); (b) personal values oriented toward security (people highly oriented toward security will show elevated worry and fear levels); (c) skepticism (COVID-19-skeptic people will have lower worry and fear levels); (d) the intention to adopt protective behaviors against COVID-19 and protective behaviors themselves, which will be positively related to worries and fears; and (e) anxiety, which is also expected to be positively related to worries and fears of COVID-19.

As there is evidence of the relationship of personal values oriented toward security with risk perception [28–30] and with protective behaviors against COVID-19 [23, 24] and anxiety [31], and as there is evidence of the relationship of risk perception with protective behaviors [26, 27] and anxiety [27], we expect that worries and fears of COVID-19 will mediate the relationship that personal values oriented toward security establish with protective behaviors against COVID-19 (H5a) and anxiety (H5b).

## Methods

### Procedure

The study comprises two phases. In Phase 1, an online questionnaire was disseminated to Spanish residents just before Christmas, between 15 and 22 December 2020, *via* the snowball sampling method. During this period, COVID-19 cases were slightly increasing and the Government and the media were giving out worrying information regarding the plausibility of a rebound in cases over Christmas by appealing to responsibility and maintenance of the utmost caution during the holidays.

In contrast, Phase 2 took place 6 months later, between 16 May and 6 June 2021, a period in which COVID-19 cases were slightly decreasing and the Government and the media were disseminating optimistic information regarding a supposed return to near-normality due to the massive increase in people getting vaccinated in Spain.

The study was conducted in accordance with the Declaration of Helsinki [32] and received the approval of the ethics committee of the University of Cordoba (Spain) through code CEIH-22-4. In both phases, before completing the questionnaire, all participants gave their informed consent.

### Participants

The questionnaire was completed by 593 participants (64.8% women; age range = 18–80 years, *M* = 42.52, *SD* = 13.82) in Phase 1 and by 328 participants (51.8% women; age range = 15–89 years, *M* = 36.95, *SD* = 15.53) in Phase 2. In Phase 2, 89 of the 328 participants were the same as in Phase 1, forming a longitudinal sample (63% women; age range = 18–69 years, *M* = 44.71; *SD* = 11.69). Further details are given in Supplementary Table S1.

### Measures

The following measures were used (Cronbach’s alpha values are shown in Table 1) and all factors were rated on a seven-point Likert scale where 1 = “Strongly disagree” and 7 = “Totally agree.” No missing data were observed, except in the intention to adopt protective behaviors related to COVID-19 transmission. In this case, the missing data were replaced by the mean for the confirmatory factorial analyses (CFA) and multigroup CFA (MGCFA) performed.

**TABLE 1:** Correlations, means, standard deviations and Cronbach’s alpha values of the studied variables in each data collection (Study Attitudes, behaviors, and psychological health in time of pandemic, Spain, 2021).

	1	2	3	4	5	6	7	8	9	10	Mean	SD	α
1. CoV-WoFe	—	0.86***	0.89***	0.15***	0.16***	−0.20***	0.52***	0.26***	0.35***	—	**3.99**	**1.43**	**0.87**
2. CoV-Wo	0.88***	—	0.52***	0.15***	0.15***	-0.26***	0.57***	0.29***	0.43***	—	**5.37**	**1.55**	**0.86**
3. CoV-Fe	0.84***	0.48***	—	0.12**	0.13**	-0.09*	0.35***	0.16**	0.19***	—	**2.61**	**1.73**	**0.89**
4. Vulnerable to COVID-19	0.12*	0.15**	*0.05*	—	0.12**	−0.10*	**0.06**	**−0.05**	**0.03**	—	—	—	—
5. Death from COVID-19	*0.10*	*0.07*	0.11*	*0.09*	—	−0.09*	0.09*	0.12*	**0.05**	—	—	—	—
6. Skepticism	—	—	—	—	—	—	−0.22***	−0.22***	−0.21***	—	**2.15**	**1.36**	**0.90**
7. Security SVO	—	—	—	—	—	—	—	0.35***	0.52***	—	**5.60**	**1.36**	**0.79**
8. Behavioral intention	0.35***	0.42***	0.16**	*0.03*	-0.11*	—	—	—	0.54***	—	**4.05**	**1.38**	**0.82**
9. Protective behavior	0.42***	0.54***	0.17**	*0.10*	*-0.09*	—	—	0.61***	—	—	**5.64**	**1.44**	**0.94**
10. Anxiety	0.29***	0.14**	0.38***	*0.06*	*0.10*	—	—	***−*** *0.04*	*−0.01*	—	—	—	—
Mean	*3.63*	*4.85*	*2.41*	—	—	—	—	*4.24*	*5.21*	*2.97*	—	—	—
SD	*1.46*	*1.79*	*1.59*	—	—	—	—	*1.32*	*1.63*	*0.98*	—	—	—
Cronbach’s alpha (*α*)	*0.87*	*0.89-*	*0.86*	—	—	—	—	*0.81*	*0.94*	*0.86*	—	—	—

*Note:* ***p < 0.001, **p < 0.01, *p < 0.05. Upper triangle values (in bold) are for the Christmas sample (first phase), and lower triangle values (in italic) are for the Summer sample (second phase); CoV-WoFe, COVID-19 Worries and Fears Scale; CoV-Wo, COVID-19-related worries for health; CoV-Fe, self-reported physiological symptoms associated with fear; SVO, social value orientation.

#### The COVID-19-Related Worries and Fears Scale (CoV-WoFe)

A short scale was created by focusing on two different expected factors [1]: CoV-Wo—worry about catching the virus and the perceived health implications of the COVID-19 consequences; and [2] CoV-Fe—the extent to which individuals feel afraid about getting COVID-19 by showing uncontrollable physiological symptoms of fear and anxiety associated with COVID-19.

The CoV-WoFe (see Supplementary Table S2) was based on the Fear of COVID-19 Scale (FCV-19S) [15] and the danger and contamination fears factors of the COVID Stress Scales (CSS) [16]. As it can be observed on Supplementary Table S2, the four Items expected to conform the CoV-Wo were designed by adapting into Spanish some Items of the CSS [16]. Moreover, the four Items expected to conform the CoV-Fe were designed by adapting into Spanish some Items of the FCV-19S [15]. The resultant scale consisted of eight items: four expected to load in CoV-Wo and four in CoV-Fe.

#### Direct Experience With COVID-19

Participants were asked whether they or someone who lives with them has a disease that makes them especially vulnerable to COVID-19 and whether they had lost a person close to him/her who has died due to COVID-19.

#### Orientation to Self-Protection

In the first data collection, to measure to what extent participants had personal values oriented toward self-protection, they responded to three items of the security factor of the Portrait Values Questionnaire [33].

#### Skepticism

In the first data collection, to measure skepticism towards the disease and the pandemic, a six-item *ad hoc* scale (see Supplementary Table S3) was designed by adapting to the pandemic situation and expanding the scale of skepticism towards climate change [34]. Participants indicated the degree to which they were skeptical about the disease and the pandemic. Exploratory factor analysis retained one unique factor that explains 67.44% of the variance.

#### Intention to Adopt Protective Behaviors Related to COVID-19 Transmission

In both phases, intention to adopt protective behaviors to prevent COVID-19 transmission was measured with the Protective Behavioral Intention against COVID Scale [23]. Participants indicated to what extent they had the intention to perform different protective behaviors to prevent virus transmission in their family gatherings (lunches and dinners) during the next Christmas period for the first sample (phase 1) and in their family and friends’ gatherings during the next Summer vacation for the second sample (phase 2).

#### Protective behaviors

In both phases, to measure to what extent participants adopted protective behaviors to prevent COVID-19 transmission, they completed the Protective Behavior against COVID Scale [23].

#### Anxiety

On the second data collection, anxiety was measured through the anxiety factor of the validated Spanish version of the Hospital Anxiety and Depression Scale [35].

### Statistical Analyses

The phase 1 sample was randomly divided into two samples of the same size. With the first split sample we explored the psychometric properties of the items to assess their suitability for inclusion in further analysis. Exploratory factor analysis (EFA) with Oblimin direct rotation was performed with the first split sample and CFA with the second split sample [36]. To explore the consistency of the expected bifactorial structure over time, CFA was also performed with the phase 2 sample.

The external validity of the CoV-WoFe was explored by assessing the correlation of the scale and its factors with other theoretically related psychosocial variables for the phases 1 and 2 samples. Moreover, with the longitudinal sample, mediation analyses were performed using the sixth model of the Process for SPSS macro (Hayes, 2013), with a confidence interval of 95% and resampling with 10,000 bootstrap samples; security orientation (Time 1) was introduced as the independent variable, CoV-Wo and CoV-Fe (Time 2) as serial mediating variables and protective behaviors against COVID-19 and anxiety (Time 2) as dependent variables.

Gender invariance was explored through MGCFA using the larger phase 1 sample. The goodness-of-fit indices (CFI, TLI and RMSEA) were assessed using the rules of thumb recommended by Schermelleh-Engel et al. [37] and the culture invariance was assessed by ∆CFI in deciding the best-fitting model, assuming that ∆CFI >0.01 indicates a reliable difference between the fitting of constrained and unconstrained models. Again, with the larger phase 1 sample, the potential differences between men and women in the scale and its subdimensions were observed through analysis of variance (ANOVA). Finally, evolution of the scale and its subdimensions was explored through repeated-measures analysis (RMA) with the longitudinal sample.

## Results

### Phase 1: Reducing and Refining the Items

#### Reliability Analyses and Correlations

The reliability level was high for the CoV-WoFe (*α* = .86) and for each one of its two expected factors (α_CoV-Wo_ = .86; α_CoV-Fe_ = 0.85). No items showed low item-to-total correlation or decreased Cronbach’s alpha if removed. Moreover, no item displayed poor correlation with half (or more) of the other items in the scale or in the factor they were expected to load; nevertheless, items 2 and 3 displayed poor correlation with all the items of the expected fear factor of the scale (CoV-Fe). Therefore, items 2 and 3 were removed due to a poor correlation with all the items of the second expected factor of the scale. This could be due to the subject of both items. While all the other items referred to the worries of the impact of COVID-19 on one’s own health, items 2 and 3 referred to the impact for loved ones. Once items 2 and 3 were deleted, the reliability level remained high for the CoV-WoFe (*α* = 0.85) and for the fear factor CoV-Wo (*α* = 0.81).

#### Exploratory Factor Analysis

EFA showed the two expected factors with a balanced factorial structure (Supplementary Table S4) that explained 77.99% of the variance, thus confirming H1. Factor 1 corresponded to CoV-Fe and Factor 2 to CoV-Wo. All the items loaded in the expected factors, except for item 5, which finally loaded in Factor 2.

### Phase 2: Validity, Reliability and Invariance of the Scale

#### Confirmatory Factor Analyses

**FIGURE 1:**
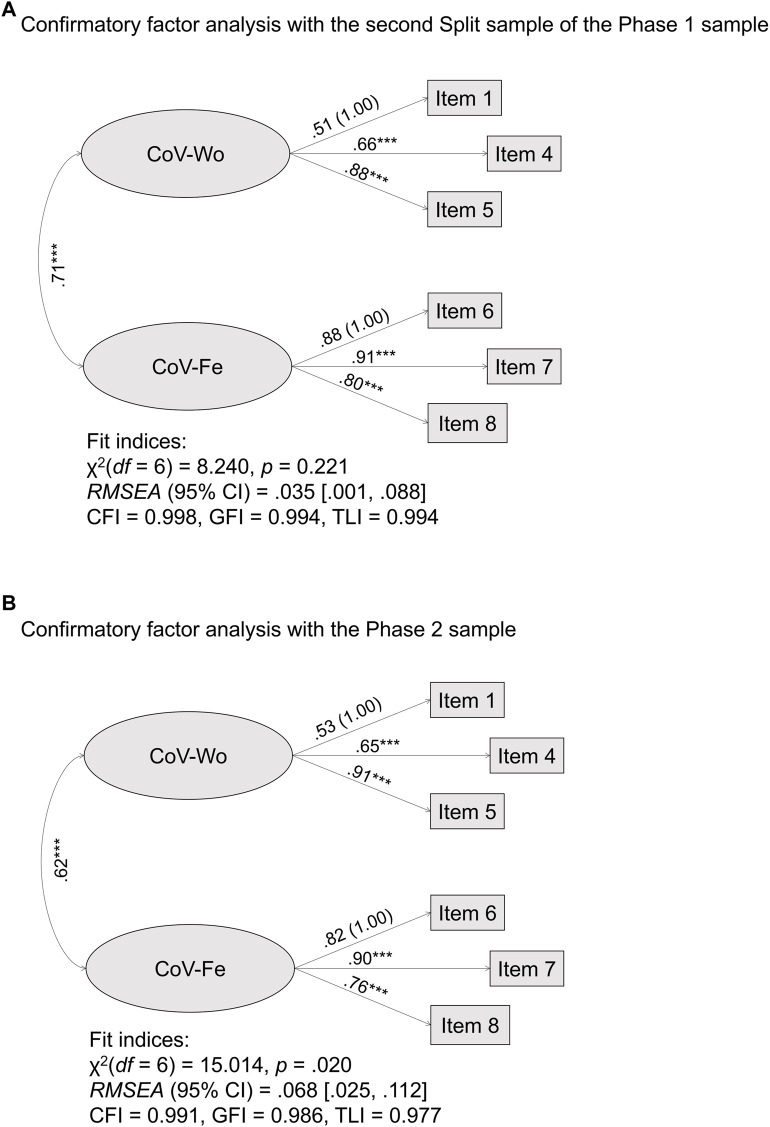
Confirmatory factor analyses of the COVID-19 Worries and Fears Scale (Study Attitudes, behaviors, and psychological health in time of pandemic, Spain, 2021). **(A)** Confirmatory factor analysis with the second Split sample of the Phase 1 sample. **(B)** Confirmatory factor analysis with the Phase 2 sample.

When performing CFA by introducing the bidimensional model found in EFA, good to excellent fit indices emerged with both the second split sample of the phase 1 sample and the phase 2 sample (Figure 1). Therefore, consistency throughout time of the expected bifactorial structure of the CoV-WoFe was confirmed, giving additional support for H1.

#### Gender Perspective

As shown in Table 2, the MGCFA results showed metric and scalar invariance between men and women, demonstrating that the bi-factorial scale was invariant for gender and valid for both men and women.

**TABLE 2:** Goodness-of-fit indices of the multigroup confirmatory factor analysis (Study Attitudes, behaviors, and psychological health in time of pandemic, Spain, 2021).

	χ^2^	*Df*	CFI	TLI	RMSEA	RMSEA [90% CI]	SRMR	ΔCFI	Δχ^2^
Configurational model	25.326*	12	0.993	0.982	0.043	[0.019; .067]	0.008		
Metric Invariance	29.960*	16	0.992	0.986	0.038	[0.016; .059]	0.017	0.001	4.634 (ns)
Scalar Invariance	42.886**	20	0.987	0.981	0.044	[0.026; .062]	0.016	0.005	12.926*

*Note. χ* ^*2*^ *,* chi-square test of model fit; df, degree of freedom; CFI, comparative fit index; TLI, Tucker-Lewis index; RMSEA, root mean square error of approximation; SRMR, standardized root mean square residual; ΔCFI, difference between the CFI values of two models (the tested model *minus* the baseline model); Δχ^2^, difference in χ^2^ estimates (the tested model *minus* the baseline model); **p < 0.01, *p < 0.05.

**FIGURE 2:**
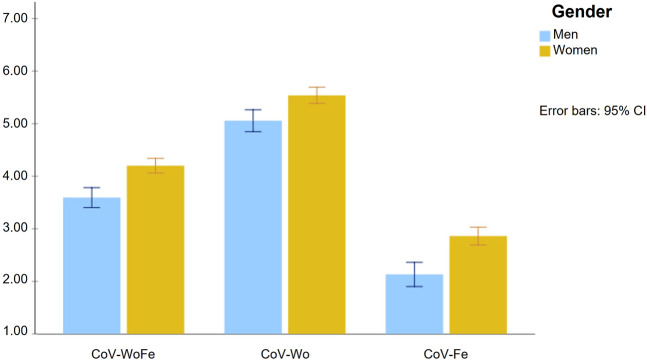
Mean differences between men and women on the COVID-19 Worries and Fears Scale and its subdimensions (Study Attitudes, behaviors, and psychological health in time of pandemic, Spain, 2021).

ANOVA (Figure 2) showed significant differences between men and women in the general CoV-WoFe [*F* (1,592) = 25.484, *p* < 0.001] and in CoV-Wo [*F* (1,592) = 13.459, *p* < 0.001] and CoV-Fe [*F* (1,592) = 25.187, *p* < 0.001], thus confirming H2.

#### Time Perspective

**FIGURE 3:**
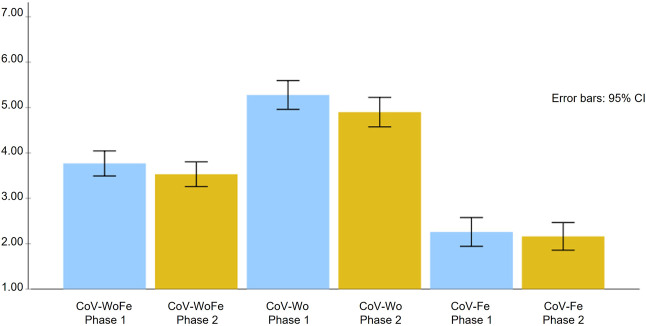
Mean and standard error of the COVID-19 Worries and Fears Scale and its subdimensions in each evaluation phase (Study Attitudes, behaviors, and psychological health in time of pandemic, Spain, 2021).

RMA (Figure 3) showed significant differences between the first and the second evaluation time in the CoV-WoFe [*F* (1, 88) = 7.06, *p* < 0.01; ɳ^2^ = 0.07, observed power (OP) = 0.75] and in CoV-Fe [*F* (1, 88) = 11.25, *p* < 0.001; ɳ^2^ = 0.11, OP = 0.91]. No differences were found in CoV-Wo [*F* (1, 88) = 0.67, *ns*; ɳ^2^ = 0.01, OP = 0.13].

#### External Validity

The expected correlations between the CoV-WoFe (and its subscales) and the other explored variables are displayed in Table 1, showing that H4 was partially supported.

The mediation analyses (Table 3) confirmed H5a and H5b: that both factors of the CoV-WoFe mediated the relationship that security orientation established with COVID-19-protective behaviors and anxiety.

**TABLE 3:** Results of the mediation analyses (Study Attitudes, behaviors, and psychological health in time of pandemic, Spain, 2021).

Protective behaviors as dependent variable (H5a)	Cov-Wo(M1)		Cov-Fe (M2)		Protective behavior (Y)	
	Coeff.	*Sd*	Coeff.	*sd*	Coeff.	*sds*
Security SVO (X)	0.50***	0.10	−0.01	0.11	0.14	0.07
CoV-Wo (M1)	—	—	0.51***	0.10	0.43**	0.07
CoV-Fe (M2)	—	—	—	—	−0.06	0.07
	Model summary	***R* ^2^ = 0.25**		***R* ^2^ = 0.26**		***R* ^2^ = 0.24**	
*F* (1, 87) = 28.57***		*F* (2, 86) = 14.73***		*F* (3, 85) = 8.75***	
Standardized indirect effect		X→M1→Y		X→M2→Y		X→M1→M2→Y	
Bootstrapp (95% CI)	0.212 [0.067, .390]		0.001[−0.022, 023]		−0.015[−0.074, 0.030]	

Anxiety as dependent variable (H5b)	CoRPS-8 F1 (M1)		CoRPS-8 F2 (M2)		Anxiety (Y)	
	Coeff.	*Sd*	Coeff.	*sd*	Coeff.	*sds*
Security SVO (X)	0.50***	0.10	−0.01	0.11	0.18	0.07
CoW (M1)	—	—	0.51***	0.10	−0.09	0.07
CoF (M2)	—	—	—	—	0.55***	0.07
	Model summary	***R* ^2^ = 0.25**		***R* ^2^ = 0.26**		***R* ^2^ = 0.33**	
*F* (1, 87) = 28.57***		*F* (2, 86) = 14.73***		*F* (3, 85) = 14.05***	
Standardized indirect effect		X→M1→Y		X→M2→Y		X→M1→M2→Y	
Bootstrapp (95% IC)	−0.042 [−0.180, .067]		−0.005 [−0.123, 0.085]		0.141 [0.060, 0.258]	

*Note*: CoV-WoFe, COVID-19 Worries and Fears Scale; CoV-Wo, COVID-19-related worries for health; CoV-Fe, self-reported physiological symptoms associated with fear; SVO, social value orientation; X, dependent variable; M, mediator; Y, independent variable; CI, confidence interval; Coeff., coefficient; SD, standard deviation.

## Discussion

This study set out to construct and validate the CoV-WoFe as a short and easy-to-apply gender-invariant tool with optimal psychometric properties to measure two different factors related to the affective dimension of COVID-19 risk perception: worries about the health consequences associated with COVID-19 (CoV-Wo) and perception of physiological symptoms associated with the fear of COVID-19 (CoV-Fe). Its development was based on other previous larger but slightly different instruments [15, 16]. The results supported a bidimensional structure with excellent fit indices that was consistent over time, coherent with the initial theoretical approach. The relevance of the CoV-WoFe relies on the possibility of measuring these two dimensions together in a brief six-item easy-to-apply instrument, in contrast to previous interesting but larger scales that measure only one of these aspects. The internal consistency indicators evaluated are optimal, supporting both the use of the two factors separately or jointly.

### External Validity of the CoV-WoFe

Coherent relationships between the CoV-WoFe (and its dimensions) and other variables of its nomological network were found, giving support to the external validity of the CoV-WoFe.

#### Personal Experience Related to COVID-19

Direct and indirect experiences of COVID-19 are factors that influence COVID-19-related worries and fears [8]. To know a close person who has died from COVID-19 supposes an increase of both CoV-Wo and CoV-Fe. Nevertheless, such an experience was positively related to CoV-Wo only in the Christmas period (phase 1), in which the social context was riskier (increasing infection and death rates) and accompanied by authorities’ cautionary messages related to self-protection and self-isolation. Non-etheless, in the second phase, during the Summer period—characterized by decreasing infection rates and authorities’ reassuring messages—the direct experience of having loved someone who has died from COVID-19 still influences CoV-Fe but not CoV-Wo. This seems logical: even in a less risky period, the traumatic experience of losing loved ones from COVID-19 may still exacerbate the more uncontrollable physiological symptoms associated with fear and anxiety. Nevertheless, general worries about the health consequences of COVID-19 may be more dependent on the health context of the pandemic situation.

In the same way, CoV-WoFe and CoV-Wo were consistently associated with being particularly vulnerable to COVID-19 or living with someone who was particularly vulnerable to it. This relation is coherent with the theoretical expectation, as particularly risky health situations of individuals or their loved ones are inevitably associated with increases in the worry over the health consequences of COVID-19. Nevertheless, CoV-Fe was associated with this variable only in the Christmas sample (first phase). These data could be coherent, considering the context of the Christmas period, with increasing COVID-19 infection and death rates and a social communication setting oriented to persuade people to maintain precautionary measures and restrict family gatherings; such factors could particularly increase fear and anxiety regarding COVID-19 and the associated physiological symptoms in people who are (or live with someone who is) particularly vulnerable to COVID-19. Nevertheless, in the second phase, during the Summer period (with decreasing infection and death rates, increasing vaccination rate and media communications transmitting confidence about the end of the health crisis in Spain), the association between being or knowing someone particularly vulnerable to COVID-19 and uncontrollable psychological symptoms related to fear is not so prominent.

#### Beliefs and Values

Coherent with previous literature showing that social value orientation is relevant in shaping risk perception [2, 29, 30], the more individuals were oriented to security, the more they were worried about the potential health consequences of COVID-19 and the more they reported physiological symptoms associated with fear of COVID-19. These relations give support to the external validity of the scale: high COVID-19 worries and fears in people with high personal security values are coherent with their self-protective orientation [33].

Moreover, congruent with previous research [25], the more individuals presented elevated scores on skepticism regarding COVID-19, the lower were their CoV-Wo and CoV-Fe levels. Clearly, for people who deny the existence and virulence of COVID-19, to be worried about its possible consequences and to suffer physiological symptoms related to fear makes no sense.

#### Protective Intentions and Behaviors

Congruent with previous research in the pandemic context [38], individuals with higher COVID-19-related worries and fears, who were especially worried about the potential COVID-19 health consequences (CoV-Wo) and report more physiological symptoms related to fear associated with COVID-19 (CoV-Fe), presented higher intentions to protect themselves from COVID-19 contagion and adopted more COVID-19-related protective behaviors.

#### Anxiety

Previous research has demonstrated that anxious individuals overestimate the probability of negative events and are prone to perceive more subjective risks for future events [39]; but also, the perception of high risk will increase anxiety by activating an anxiety state [27, 40]. In the pandemic context, it has been demonstrated that the more individuals perceive the COVID-19 situation as risky, the more they feel anxious [16]. In our research, the CoV-WoFe and its two subdimensions were positively associated with anxiety. The more individuals perceive high health-related risk associated with COVID-19 and the more they perceive symptoms related to fear, then the more they feel anxious (H4e). Moreover, the factor of the CoV-WoFe that highly correlated with anxiety was the fear component. Given that anxiety symptoms are related to somatic states that are referenced in the symptoms related to the fear factor of the CoV-WoFe, this higher association with anxiety of CoV-Fe in comparison with CoV-Wo gives additional evidence for the bifactorial structure of the scale.

#### Mediating Role of the COVID-19-Related Worries and Fears

Congruent with the previous literature [23, 24, 26–31] and with our expectation, both CoV-Wo and CoV-Fe acted as serial mediators in the relationship that self-protection values establish with both the COVID-19-protective behavior (H5a) and anxiety (H5b). This result highlights the perceptual aspect of risk as a fundamental element in the adoption of protective behaviors, as well as in subjective perceived health, and has relevant applied implications regarding the communication provided to people during the pandemic: an accurate communication adapted to the situation that reflects the real risk of the pandemic state is necessary for people to adopt the appropriate self-protective behaviors; moreover, the appropriateness and adjustment of the risk communication is also relevant, as overestimated risk communication could increase the anxiety levels of the population, which have been significantly increased already as a consequence of the pandemic situation [41].

### Gender Perspective

The CoV-WoFe has been shown to be gender-invariant, highlighting the possibility of using it for both genders in the research and professional fields, with valid results. Moreover, it responds to the need to conduct research in the COVID-19 field from a gender perspective [17].

When analyzing the scores of men and women, significant differences were found. Women scored higher on risk perception in general and in each one of the two observed dimensions (H2), congruent with previous research [19, 20], providing additional evidence for the validity of the construct. These gender differences are particularly relevant if we consider that overestimation of the risk of COVID-19 could suppose higher anxiety levels, which is the case in women during the pandemic [42–45], therefore psychological interventions oriented to decrease COVID-19-related anxiety levels should consider the risk perception level of women in particular.

### Time Perspective

Significant differences were found between the first and second evaluation periods in the CoV-WoFe and in the perceived risk for health (CoV-Wo), with lower levels of COVID-19 risk perception in the second evaluation period (Summer). These results are congruent with the expectation that COVID-19-related worries will be lower in a period in which the infection and death rates are decreasing and the risk communication from authorities and media is reassuring and optimistic (H5). Thus, the fact that CoV-Wo is higher in the first phase (Christmas) is consistent with the real risk and that informed by the authorities and media, considering the contagion rates and the concurrent health circumstances. Nevertheless, no differences were found in CoV-Fe, which suggests that the physiological aspects associated with fear may be less context dependent and more anchored in individual variables.

### Limitations and Future Research

Among the possible limitations of this research are the sampling method and the small sample size of the longitudinal study. Moreover, all the variables were not included in both measurement times. This precludes the possibility of clarifying reciprocal influences between indeterminate and determined variables. Given this limitation and the limited sample size, it was not possible to perform a cross-lagged panel model. Future research should replicate the relationships found in the present study with a larger sample size and a more powerful cross-lagged panel model.

Another limitation concerns the analyses and findings resulting from the longitudinal data. The level of experimental death was very high. Moreover, some differences were found in certain variables: in comparison to participants who decided to drop out of the study, participants who decided to continue in the second measurement phase showed lower levels of CoV-Fe and skepticism and higher levels of protective behavior in the first measurement phase (but no differences were found in CoV-Wo, security orientation or intention to adopt protective behavior). Although the differences found are congruent—because people who feel more engaged in a study about COVID-19 could be people who perceive that this illness exists (with a lower level of skepticism) and thus engage in protection, but they could also be people who do not express anxiety symptoms when thinking about this illness (CoV-Fe)—this can be a problem regarding generalization of the results that use longitudinal data. In this sense, the results of the mediational analyses should be taken with precaution, as the longitudinal sample is prone to selection bias. In this regard, future research should replicate the findings on mediation resulting from the analyses performed with the longitudinal data of the present study.

In future research, it would be convenient to continue investigating the relationships between real and perceived risk and the contributing factors. It could also be interesting to adapt this brief scale to other extreme situations or catastrophes, to be used as a versatile measure in other types of critical scenarios.

### Conclusion

A brief, reliable, gender-invariant bidimensional scale (the CoV-WoFe) applicable in different samples and scenarios was validated. The two dimensions reflected the worry associated with COVID-19 health consequences (Factor 1) and the self-perceived physiological symptoms associated with fear (Factor 2). The internal consistency and validity indicators suggest that this is a very short, optimal instrument that can be used in health and psychosocial research with sufficient psychometric guarantees for both genders.
