# Supplementary material for: Development and Validation of the COVID-19 Worries and Fears Scale
Source: Int J Public Health. 2023 Jan 9;67:1604600. doi: 10.3389/ijph.2022.1604600 (PMC9868128; doi:10.3389/ijph.2022.1604600)
Supplement: Supplementary file 1 [file Table1.DOCX]

| **Supplementary Table 1**  *Sociodemographic characteristics of the participants (Study Attitudes, behaviors, and psychological health in time of pandemic, Spain, 2021).* | | | | | | | | | | | |
| --- | --- | --- | --- | --- | --- | --- | --- | --- | --- | --- | --- |
|  | Phase 1 sample | |  | Phase 2 sample | |  | Longitudinal sample | | | | |
|  |  |  |  |  |  |  | Phase 1 | |  | Phase 2 | |
|  | Valid % | *M* (*sd*) |  | Valid % | *M* (*sd*) |  | Valid % | *M* (*sd*) |  | Valid % | *M* (*sd*) |
| Gender |  |  |  |  |  |  |  |  |  |  |  |
| Female | 64.8 | - |  | 51.8 | - |  | 63.0 | - |  | 63.0 | - |
| Male | 35.2 | - |  | 48.2 | - |  | 37.0 | - |  | 37.0 | - |
| Age (years) | - | 42.52  (13.82) |  | - | 39.95 (15.53) |  |  | 44.71 (11.69) |  |  | 44.71 (11.69) |
| Reported not having or not living with someone who has an illness or chronic health problem that makes them especially vulnerable to COVID-19 | 60.5 | - |  | 61.3 | - |  | 57.5 | - |  | 52.8 | - |
| Reported that a family member or close friend had died from COVID-19 | 28.2 | - |  | 26.5 | - |  | 31.5 | - |  | 37.1 | - |
| Vaccine schedule |  |  |  |  |  |  |  |  |  |  |  |
| Reported not having received any dose of the vaccine | - | - |  | 66.0 | - |  | - | - |  | 73.0 | - |
| Reported having received an incomplete vaccine schedule | - | - |  | 23.5 | - |  | - | - |  | 19.0 | - |
| Reported having received a complete vaccine schedule | - | - |  | 10.7 | - |  | - | - |  | 8.0 | - |
