# Supplementary material for: Development and Validation of the COVID-19 Worries and Fears Scale
Source: Int J Public Health. 2023 Jan 9;67:1604600. doi: 10.3389/ijph.2022.1604600 (PMC9868128; doi:10.3389/ijph.2022.1604600)
Supplement: Supplementary file 2 [file Table2.DOCX]

| **Supplementary Table 2**  *Spanish and English items of the COVID-19 Worries and Fears Scale (Study Attitudes, behaviors, and psychological health in time of pandemic, Spain, 2021).* | | |
| --- | --- | --- |
| Spanish items | English items | Item design |
| 1. Me preocupa contagiarme del COVID | I am worried about catching COVID | Adapted from item 1 of the CSS (Taylor et al., 2020) |
| *2. Me preocupa que mi familia se contagie del COVID | I am worried that my family will get COVID | Ad-hoc item |
| *3. Me preocupa que el sistema de salud no pueda protegerme a mí o a mis seres queridos del COVID | I am worried that the healthcare system won’t be able to protect me and my loved ones from COVID | Combination of the adaptation of items 3 and 4 of the CSS scale (Taylor et al., 2020) |
| 4. Me preocupa que las medidas básicas (lavarme las manos, ventilar, mantener el distanciamiento social, etc.) no puedan mantenerme a salvo del COVID | I am worried that basic measures (handwashing, ventilating, maintaining social distancing, etc.) can’t keep me safe from COVID | Combination of the adaptation of items 5 and 6 of the CSS scale (Taylor et al., 2020) |
| 5. Tengo miedo al COVID | I am afraid of COVID | Adapted from item 1 of the FC-19S (Ahorsu et al., 2020) |
| 6. No duermo bien porque contagiarme del COVID me preocupa | I don't sleep well because getting COVID worries me | Adapted from item 9 of the FC-19S (Ahorsu et al., 2020) |
| 7. Mi corazón late deprisa cuando pienso acerca de la posibilidad de contagiarme del COVID | My heart beats fast when I think about the possibility of catching COVID | Adapted from item 10 of the FC-19S (Ahorsu et al., 2020) |
| 8. Cuando veo noticias o historias sobre el COVID en los medios me pongo nervioso o angustiado | When watching news and stories about COVID on social media, I become nervous or anxious | Translated from item 8 of the FC-19S (Ahorsu et al., 2020) |
| *Note:* CSS = COVID Stress Scale; FC-19S = Fear of COVID-19S  *Item eliminated from the final scale | | |
