# Supplementary material for: Development and Validation of the COVID-19 Worries and Fears Scale
Source: Int J Public Health. 2023 Jan 9;67:1604600. doi: 10.3389/ijph.2022.1604600 (PMC9868128; doi:10.3389/ijph.2022.1604600)
Supplement: Supplementary file 3 [file Table3.DOCX]

| **Supplementary Table 3**  *The pandemic and the COVID-19 Skepticism Scale (Study Attitudes, behaviors, and psychological health in time of pandemic, Spain, 2021).* | | | | | | | |
| --- | --- | --- | --- | --- | --- | --- | --- |
|  | | | | | | | |
| Coronavirus-19 supposed a real risk, or it has been exaggerated? Please indicate if you agree with those sentences by using the proposed scale (1 = Absolutely disagree; 7 = Absolutely agree). | | | | | | | |
| The media is exaggerating the COVID problem | 1 | 2 | 3 | 4 | 5 | 6 | 7 |
| Doctors and experts are exaggerating the magnitude of the COVID problem | 1 | 2 | 3 | 4 | 5 | 6 | 7 |
| COVID is not as serious as it is being reported through the different media | 1 | 2 | 3 | 4 | 5 | 6 | 7 |
| The media is often too alarmist about the COVID problem | 1 | 2 | 3 | 4 | 5 | 6 | 7 |
| I do not believe that COVID is as important a health problem as they want us to believe | 1 | 2 | 3 | 4 | 5 | 6 | 7 |
| Many leading experts still question the importance of COVID | 1 | 2 | 3 | 4 | 5 | 6 | 7 |
|  |  |  |  |  |  |  |  |
