# Supplementary material for: Development and Validation of the COVID-19 Worries and Fears Scale
Source: Int J Public Health. 2023 Jan 9;67:1604600. doi: 10.3389/ijph.2022.1604600 (PMC9868128; doi:10.3389/ijph.2022.1604600)
Supplement: Supplementary file 4 [file Table4.DOCX]

| **Supplementary table 4**  *Results of exploratory factor analysis of the COVID-19 Worries and Fears Scale: Factor loading and reliability estimates (Study Attitudes, behaviors, and psychological health in time of pandemic, Spain, 2021).* | | |
| --- | --- | --- |
|  | Highest loading for each one of the two factors | |
| Items | **Factor 1:** Worries about health consequences (CoV-Wo) | **Factor 2:** Physiological symptoms associated with fear (CoV-Fe) |
| Item 1 | -0.077 | **0.907** |
| Item 4 | -0.025 | **0.853** |
| Item 5 | 0.187 | **0.759** |
| Item 6 | **0.908** | 0.002 |
| Item 7 | **0.960** | -0.043 |
| Item 8 | **0.841** | 0.058 |
| **Scale reliability estimates** |  |  |
| Cronbach's alpha values | 0.81 | 0.89 |
| Percentage of explained variance | 57.02 | 20.97 |
| **Suitability for the EFA use** | | |
| Kaiser-Meyer-Olkin index | 0.792 | |
| Bartlett’s test of sphericity | χ²=929.477; *df* = 15; *p* < .001 | |
